# Exploration of Rapid Evaporative-Ionization Mass Spectrometry as a Shotgun Approach for the Comprehensive Characterization of *Kigelia Africana* (Lam) Benth. Fruit

**DOI:** 10.3390/molecules25040962

**Published:** 2020-02-20

**Authors:** Katia Arena, Francesca Rigano, Domenica Mangraviti, Francesco Cacciola, Francesco Occhiuto, Laura Dugo, Paola Dugo, Luigi Mondello

**Affiliations:** 1 Foundation A. Imbesi c/o University of Messina, I-98168 Messina, Italy; arenak@unime.it; 2 Department of Chemical, Biological, Pharmaceutical and Environmental Sciences, University of Messina, I-98168 Messina, Italy; dmangraviti@unime.it (D.M.); focchiuto@unime.it (F.O.); pdugo@unime.it (P.D.); lmondello@unime.it (L.M.); 3 Department of Biomedical, Dental, Morphological and Functional Imaging Sciences, University of Messina, I-98168 Messina, Italy; cacciolaf@unime.it; 4 Department of Sciences and Technologies for Human and Environment, University Campus Bio-Medico of Rome, I-00128 Rome, Italy; l.dugo@unicampus.it; 5 Chromaleont s.r.l., c/o Department of Chemical, Biological, Pharmaceutical and Environmental Sciences, University of Messina, I-98168 Messina, Italy; 6 BeSep s.r.l., c/o Department of Chemical, Biological, Pharmaceutical and Environmental Sciences, University of Messina, I-98168 Messina, Italy

**Keywords:** REIMS, direct infusion in mass spectrometry, mass accuracy, tandem MS, *Kigelia africana* fruit, bioactive molecules, lipids, phenols

## Abstract

Rapid evaporative-ionization mass spectrometry (REIMS) coupled with an electroknife as a sampling device was recently employed in many application fields to obtain a rapid characterization of different samples without any need for extraction or cleanup procedures. In the present research, REIMS was used to obtain a metabolic profiling of the *Kigelia africana* fruit, thus extending the applicability of such a technique to the investigation of phytochemical constituents. In particular, the advantages of REIMS linked to a typical electrosurgical handpiece were applied for a comprehensive screening of this botanical species, by exploiting the mass accuracy and tandem MS capabilities of a quadrupole-time of flight analyzer. Then, 78 biomolecules were positively identified, including phenols, fatty acids and phospholipids. In the last decade, *Kigelia africana* (Lam.) Benth. fruit has attracted special interest for its drug-like properties, e.g., its use for infertility treatments and as anti-tumor agent, as well as against fungal and bacterial infections, diabetes, and inflammatory processes. Many of these properties are currently correlated to the presence of phenolic compounds, also detected in the present study, while the native lipid composition is here reported for the first time and could open new directions in the evaluation of therapeutic activity.

## 1. Introduction

In recent years, folk medicine has attracted ever greater interest emerging as milestone in modern “alternative” medicine. Around 60% of antitumor and anti-infective drugs available on the market come from natural products [1] and many pharmaceutical industries are involved in exploring and utilizing new active principles from various plant sources, due to their prophylactic and therapeutic potential. Moreover, the World Health Organization (WHO) estimates that 80% of African, Asian and Latin American population, especially those who live in the countryside, depend on alternative medicine for their health requirements [2]. Medicinal plants are widespread in developing countries, due to no regular access to modern medicine among the rural population left with no other alternative than to use traditional medicine [3]. As a consequence, researchers are asked to evaluate the composition as well as perform safety and quality controls of plants commonly used.

Among them, *Kigelia africana* (Lam.) Benth. (Bignoniaceae), syn. *Kigelia pinnata* (Jacq.) D.C., a widespread tropical tree in Senegal, Ethiopia and in the north parts of South Africa, has recently gained particular attention for its potential drug-like properties. It is also known as cucumber or sausage tree due its huge fruits (roughly 0.6 m in length and 4 kg in weight) originating from long fibrous stalks [4]. The fruits are the part used in traditional medicine and are frequently used for infertility treatments, anti-tumor activity, for fungal and bacterial infections, diabetes, and anti-inflammatory processes [5]. Also, the fruit extracts have proved to possess antioxidant activity, which could drive the treatment of liver-borne disease [6]. 

The medicinal properties of *Kigelia africana* can be traced back to the presence of numerous secondary metabolites. Previous phytochemical studies reported the presence of iridoids, flavonoids, triterpenes, fatty acids, coumarins, quinones, steroids and lignans [7,8,9,10,11,12]. For these evaluations different analytical methods have been developed according to the nature of the analytes taken into consideration. As a consequence, only specific classes of compounds were monitored in each analysis. Polyphenols and intact lipids (phospholipids PLs and triglycerides TGs) are usually analysed by high-performance liquid chromatography (HPLC), after a proper sample preparation procedure which significantly increase both analytical cost and the total analysis time. Sterols and fatty acids (FAs) are elucidated by the gas chromatography (GC) technique, which, in addition to a lipid extraction and/or saponification step, requires a derivatization process, namely the conversion of the analytes into more volatile and less polar compounds, before the injection. 

In this work, for the first time full metabolomics work was carried out by a shotgun mass spectrometry (MS) approach, which exploits an ambient ionization MS source, which vaporize and ionize molecules directly from the sample, without any sample pre-treatment. In particular, the rapid evaporative-ionization mass spectrometry (REIMS) source [13,14,15,16,17] linked with an electrosurgical hand piece [18,19,20] was employed. This innovative technique allows us to obtain an immediate profiling of different samples without any requirement of sample preparation or pre-chromatographic separation. The analyses were carried out in only 3-4 sec, thanks to the thermal ablation of the sample with the electroknife, which generates an aerosol with molecular vapors inside, ionized and detected by a specific MS system. In the present work a hybrid MS detector was employed, based on a quadrupole-time of flight (Q-ToF) analyzer, capable of generating mass accuracy data, directly searchable in commercial and online database, and tandem MS (MS/MS) experiments for structure elucidation purposes. An untargeted screening of the fruit of *Kigelia africana* was carried out leading to the identification of the main phytochemicals constituents, such as phenolic compounds, FAs, PLs and TG. In addition, conventional chromatography techniques were deployed to confirm peak identification and demonstrate the robustness of the new technique in the detection of compounds belonging to different chemical families. 

## 2. Results and Discussion

### 2.1. Mass Spectral Data

To the best of our knowledge, this is the first work dealing with the untargeted characterization of a botanical species by the novel REIMS technology, taking also into account that the majority of the applications reported in the literature focus on animals [17,20,21,22,23,24,25,26] and microorganisms [27,28,29,30], apart from several studies involving human cancer [14,15,18,20,31,32]. 

**Figure 1:**
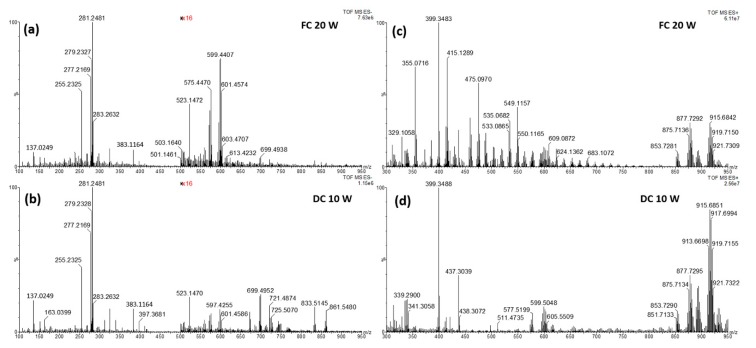
(**a**) Rapid evaporative-ionization mass spectrometry (REIMS) (-) spectrum over the mass range 100–950 *m/z* of a cut of *K. africana*, in forced-coagulation FC mode at a power of 20 W; (**b**) REIMS (-) spectrum over the mass range 100–950 *m/z* of a cut of *K. africana*, in dry-cut (DC) mode at a power of 10 W; (**c**) REIMS (+) spectrum over the mass range 300–950 *m/z* of a cut of *K. africana*, in FC mode at a power of 20 W; (**d**) REIMS (+) spectrum over the mass range 300–950 *m/z* of a cut of *K. africana*, in DC mode at a power of 10 W.

As a consequence, the first object was the development of a suitable analytical method able to maximize the system performances. Among all the analytical parameters involved, e.g., mass range, event time, cone voltage and frequency applied to the electroknife, the last results in being strongly related to the signal intensity obtained. As a matter of fact, it determines the vaporization of the analytes from the matrix, then it is essential for the next step. Given the dried nature of the sample, and its low intrinsic electrical conductivity, the powerful forced-coagulation (FC) and dry-cut (DC) modes were tested to create a visible burn mark, despite the first one is normally discouraged due to the extensive damage of the sample [33]. Figure 1 reports the comparison between MS spectra obtained in FC and dry cut (DC) mode, in both positive and negative ionization mode.

It can be highlighted that the FC mode produced a very noisy spectrum in negative ionization, hampering the detection of high molecular weight compound, also characterized by a very low signal intensity. Then the DC mode was selected for the analysis in negative. 

By contrast, in positive ionization the spectra obtained in FC mode were more informative than those acquired in DC mode, mainly polyphenols, as confirmed during the identification process. A quantitative comparison is reported in Tables S1 and S2, which contain the signal-to-noise (S/N) ratios of ions spread over the mass range in negative and positive ionization mode, respectively. The S/N ratio was calculated by dividing the ion intensity for the average intensity of the background in the spectral region of the specific ion. The selected ions are those for which a putative assignment was performed. In negative ionization mode, the DC at 10 W produced the highest S/N ratio for all the ions mainly due to a lower noise compared to the FC mode at 20 W. In positive ionization, the FC mode at 20 W produced in most cases the highest S/N ratio, mainly due to a highest signal intensity, despite a more intense noise that sometimes significantly reduces the S/N values, such as in the case of the ions at *m/z* 339.2898. Both tables highlight that some ions are detected only by using the selected power of cutting; specifically, phospholipids are totally lost by setting the diathermy generator in FC mode at 20 W and some phenols are not detected in positive ionization if the cut is performed in DC mode at 10 W.

However, in both cases, the use of low electrical power (10 and 20 W, respectively, in negative and positive ionization modes) resulted in the achievement of the best S/N in the entire mass range. Repeatability and reproducibility were also evaluated at both intra- and interday levels, within the same sample and two different samples collected in two different years (the coefficients of variation (CV%) of three different cuts for each day or sample was calculated). Data are shown in Table 1 for 7 representative ions covering the entire mass range in both ionization modes. Values less than 25% were obtained for all the peaks, which can be considered acceptable for an ambient MS technique. 

As for the elucidation of the qualitative profile, this kind of metabolomics studies usually involves the use of high-resolution MS systems in combination with dedicated software that match MS data with those reported in online and/or commercially available databases [34,35,36].

To this regard, mass accuracy acquires a central role in order to obtain a restricted list of candidates for each MS value. In order to verify the minimal instrumental requirements, the instrument was calibrated prior of the use by infusing a solution of ammonium formate and a mass accuracy of less than 0.5 ppm was obtained. However, the application of the electroknife directly on the sample can provide a mass error significantly larger, due to the introduction in the MS analyzer of a large amount of ions without any sample clean-up or chromatographic separation. For this reason, internal lock-mass correction was performed in each analysis against the endogenous ions *m/z* 281.2486 and *m/z* 855.7436 in negative and positive ion modes, respectively. Such an operation is mandatory prior to a database search to correct any mass drift. Then, a maximum mass error of 10 ppm, corresponding to a precision ranging between 0.01 and 0.001 Da, was set for compound identification by the Progenesis QI software. However, a mass error less than 5 ppm was calculated for the majority of the compounds (Table 2 and Table 3). Moreover, the use of both ionization mode, both positive and negative, allows to achieve a comprehensive sample profile, by the detection of compounds characterized by totally different chemical structures. In the present work, polar compounds were mainly detected in negative polarity, such as in the case of small phenolic compounds, fatty acids (FA) and phospholipids (PLs), whereas non polar species were detected in positive polarity, such as in the case of acylglycerols (mono-, di- and triglycerides) and few phenol derivatives in which the presence of ester bonds reduce the polarity and facilitate the formation of adduct with sodium or potassium ions. This can be also related to a larger chemical structure with respect to smaller phenolic compounds, e.g., benzoic acids, quinones and pyranones. 

**Figure 2:**
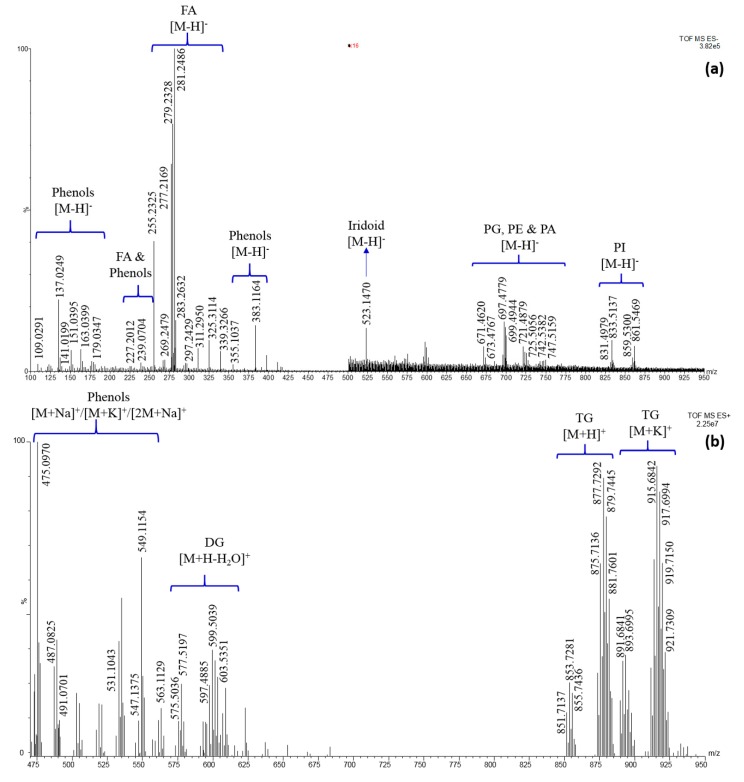
(**a**) REIMS (-) spectrum over the mass range 100–950 *m/z*—the 500–950 *m/z* region was magnified of a 16 factor; (**b**) REIMS (+) spectrum over the mass range 470–950 *m/z*. A class identification is also reported, along with the ion type detected. DG = diglyceride; FA = fatty acid; MG = monoglyceride; PA = phospatidic acid; PE = phosphatidylethanolamine; PG = phosphatidylglycerol, PI = phosphatidylinositol; TG = triglyceride.

Figure 2 shows the mass spectra acquired in both ionization mode, along with the identification of the chemical class for each MS region. It can be highlighted that only deprotonated molecules [M-H]^-^ were detected in negative ion mode, while adduct with the ubiquitous sodium and potassium ions [M + Na]^+^ and [M + K]^+^ were significantly present in the positive spectrum, together with protonated molecules [M + H]^+^ and dehydrated structures [M + H − H_2_O]^+^. Progenesis QI was used for the tentative identification of significant ions, by setting the proper mass error (10 ppm) and the minimal ion intensity to perform a suitable background subtraction. However, a very long list of candidates can be obtained for each *m/z* value, due to the presence of different compounds all providing a very low mass error. 

The choice of the most probable candidate was driven, in a first moment, by the literature data, including the information coming from an online database [34] about the most probable source of each candidate, thus excluding synthetic molecules and compound normally present in animals, humans, bacteria and fungi. Afterward, MS/MS analyses were performed for structure elucidation of selected ions and when it failed, as normally happens for small analytes, chromatographic techniques were helpful to identify the right candidate. In this case an extraction procedure was performed to isolate the analyte of interest, and then the extract was injected into the chromatographic system, followed by the injection of available standard compounds. In this case, isomers can be distinguished on the basis of different retention times.

As for quantitative considerations, in general REIMS linked to an electrosurgical handpiece, being an ambient MS method, is not suitable for quantitative analysis. In fact, the matrix effect can significantly affect method accuracy and ambient conditions have a great influence on the analysis starting from the sampling by the knife, passing through the vapor transfer to the source, up to the MS spectra acquisition. Then, only relative quantification was performed within the same chemical class, supposing that, if identical experimental conditions are applied, similar compounds have the same MS response. The area percentages of acylglycerols, FAs, PLs and phenols are provided in Table 2 and Table 3.

A more detailed discussion about each class will be provided in the next sections.

### 2.2. Phenol-Like Compounds

The biological properties of *K. africana* are mainly related to the presence of antioxidant molecules, such as iridoids, flavonoids, coumarins and quinones, already reported in the literature [5,7,37,38,39,40]. 

They are normally isolated from the sample by liquid-liquid extraction and separated by HPLC, in order to concentrate them and avoid any interference from matrix constituents. This common procedure is performed at the expense of a very long analysis time. However, not more than 10 compounds were identified in these previous works [41,42]. In addition, a very recent work demonstrated that such constituents change depending on the cultivation region of the plant [5]. In the present research, a total of 17 phenol-like compounds were detected and are reported in Table 2, along with the detected ions (deprotonated molecules for the majority of the assigned structures, but also sodium or potassium adducts were found), by using complementary information coming from negative and positive ionization. Specifically, small molecules in a mass range between 100 *m/z* and 400 *m/z* were detected mainly in negative mode as deprotonated molecules, with the exception of rosmarinic acid which was detected only in positive mode, probably due to the presence of an ester bond which facilitates the formation of a sodium adduct. Indeed, larger molecules (molecular weight >450 Da) produced an intense sodium adduct in the positive MS spectrum, as in the case of the p-coumaric ester of epigallocatechin (molecular weight 452 Da), the glycosylated flavone isoquercetrin (molecular weight 464 Da) and the iridoid verminoside (molecular weight 524 Da). The last one generated also a small signal in the negative MS spectrum. 

As for smaller molecules, MS/MS experiments provided a clean spectrum, helpful for structure elucidation purposes. Some of them are reported in Supplementary Materials (Figures S1–S7), along with fragment elucidation in the relative captions. Nevertheless, in many cases positional isomers could have a very similar fragmentation pattern, then HPLC-MS analyses were carried out to identify the right isomer on the basis of retention behavior. In particular, the injection of different isomeric structures was carried out; for instance, both 2-hydroxybenzoic acid (common name salicylic acid) and 4-hydroxybenzoic acid were injected, but only the second one properly matched with the peak of the analyte in the sample. The same strategy was adopted to exclude 1,3-benzenediol, while vanillin, vanillin alcohol, p-coumaric acid, caffeic acid, daidzein and isoquercetrin were all confirmed.

As for larger phenolic structures, MS/MS experiments generated low-intensity spectra, thus making difficult the confirmation of the candidates suggested by the library search through Progenesis QI software.

**Figure 3:**
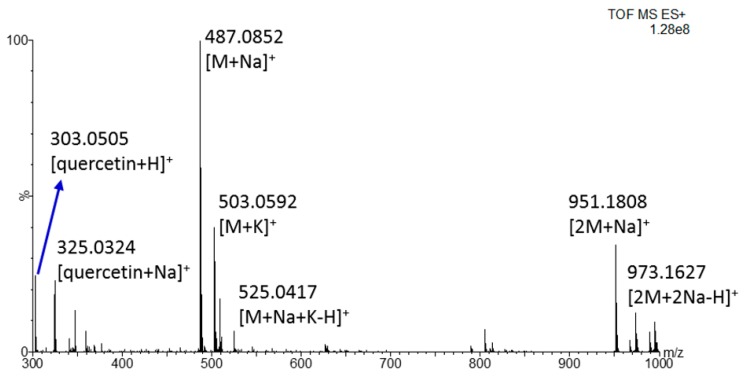
REIMS (+) spectrum, obtained by the direct infusion by the syringe pump (flow rate 50 μL/min) of isoquercetrin (1 ppm in methanol) over the mass range 300–100 *m/z*.

Taking into account the novelty of the REIMS ionization source, which most commonly focused on lipid compounds [13,14,15,16,17,18,20,21,22,23,24,25,26,31,32], some additional experiments were necessary to confirm the identification of these phenols. In particular, the polyphenol isoquercetrin, available as pure standard, was infused by the syringe pump into the REIMS source to elucidate the ionization mechanism in positive polarity. The spectrum, reported in Figure 3, highlights a very intense signal for the sodium adduct, confirming previous hypothesis. Also, the potassium adduct and the sodiate dimer were observed, thus increasing the reliability of the tentative identification of daidzein, epigallocatechin-p-coumaroate and verminoside, identified as sodiate dimer, sodium and potassium adduct with a mass error minor than 10 ppm. Within this context, the detection of more adduct ions represented a further confirmation of the proposed identity for each signal. Furthermore, the ion corresponding to the aglycon portion, in this case quercetin, was detected along with its sodiate adduct, thus pinpointing a certain degree of in-source fragmentation. This could be the reason for which the further fragmentation of the parent ions at different collision energy did not produce any intense MS/MS spectrum, especially if acquired by cutting the sample with the electroknife rather than in direct infusion mode. However, some diagnostic fragments were observed such as the ion at *m/z* 324.02 which corresponds to the loss of the glucoside portion from the sodiate adduct (Figure S8). 

Similarly, the MS/MS spectrum of the ion at *m/z* 523.15, detected in negative mode and tentatively identified as deprotonated verminoside, that is a caffeic acid derivative, produced the fragments at *m/z* 179.02 and 161.01 which could be the deprotonated and the dehydrated caffeic acid, respectively (Figure S9).

In addition, 8 compounds out of 17 were confirmed by the literature information [41,42,43], while four compounds were not present in previous reports neither confirmed by complementary techniques due to the unavailability of the pure standard. However, two of them, namely feruloyglucose and epigallocatechin-p-coumaroate were selected as tentative assignment due to their chemical structure similar to the confirmed p-coumaric and caffeic acids, which can be considered precursors of rosmarinic and ferulic acids. In addition, p-coumaric acid (or 4-hydroxycinnamic acid) is involved in the phenylpropanoid biosynthetic pathway, from which flavonoids (e.g., isoquercetrin, catechin and daidzein) are originated. Therefore, feruloylglucose and epigallocatechin-p-coumaroate could presumably be included in the list of tentative assignments. 

Piperenol A and Piperenol A triacetate are benzoyl derivatives, commonly found in herbs and spices [34], and were not reported in previous *Kigelia africana* characterization. However, the triacetate form was identified as potassium adduct with a very low mass error and was the only candidate generated from the automatic match with the Metlin, LipidMaps and Humane metabolome databases. Furthermore, the detection of the alcoholic form in negative mode could increase the probability associated to the tentative identification of both compounds. 

Finally, the relative quantification approach highlighted a high percentage of 4-hydroxybenzoic acid and piperenol A among the compounds detected in negative as deprotonated molecules, while an intense signal was obtained in positive for epigallocatechin-p-coumaroate which provided a peak area significantly larger with respect to the other sodiate adducts. The sodiate adduct of the dimer was detected only for daidzein while, among the potassium adducts, piperenol A triacetate produced the highest signal.

### 2.3. Lipid Compounds

**Figure 4:**
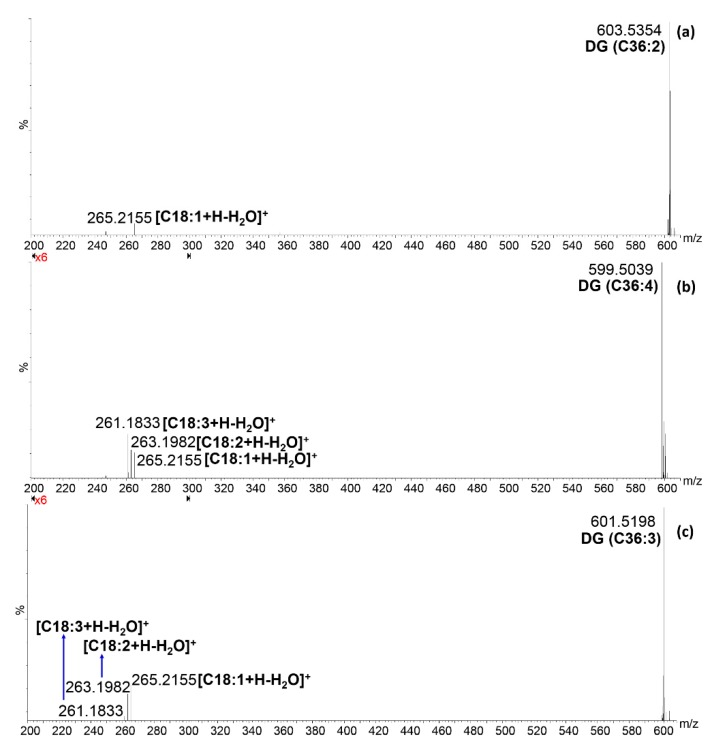
Tandem mass spectrometry (MS/MS) spectrum over the range 200–610 *m/z* of (**a**) *m/z* 603.52; (**b**) *m/z* 599.49; (**c**) *m/z* 601.50; obtained in positive ionization mode at a collision energy of 30 eV.

Conversely, from phenolic compounds, which were partially reported in the literature about this fruit, the characterization of the native lipid composition is here reported for the first time. Very recently, Gomes and co-workers [44] reported the FA profile of the *K. africana* seed oil, after the conversion of intact lipids into FA methyl esters, namely after the breaking of the ester bond of the original lipid. The qualitative profile obtained in that study is in agreement with FAs identified in the present work. In particular, as from Figure 2a and Table 3, palmitic acid (C16:0) was the most abundant saturated FA, followed by stearic acid (C18:0), heneicosanoic acid (C21:0), arachidic and docosanoic acids (C20:0 and C22:0, respectively), heptadecanoic acid (C17:0) and myristic acid (C14:0), listed according to decreasing signal intensity. Among monounsaturated FA, octadecenoic acid (C18:1) was the most abundant, followed by small amounts of hydroxy-octadecenoic acid and heptadecenoic acids (C18:1OH and C17:1, respectively), and very small levels of hexadecenoic acid and eicosenoic acids (C16:1 and C20:1, respectively). Finally, very intense signals were generated from octadecadienoic acid and octadecatrienoic acid (C18:2 and C18:3), which were the only polyunsaturated FAs detected. On the basis of these semi-quantitative considerations, it should be possible to select the most probable combination of FAs in PLs, di- and tri-glycerides (DG and TG, respectively) from the list of candidates coming from Progenesis QI. For instance, DG and PLs containing 5 and 6 double bonds most likely derive from the combination of C18:2 and C18:3 or two C18:3. Similarly, when only 1, 2 or 3 unsaturations were present in DG and PL characterized by a carbon number of 34 and 38, the combinations of C18:1, C18:2 and C18:3 with C16:0 and C20:0 could be selected as the most probable. As a consequence, only the DG and PL species in which carbon number is equal to 36 and the unsaturations range from 0 to 4 needed for MS/MS confirmatory analysis. Figure 4 shows the MS/MS spectra for DG (C36:2), DG (C36:3) and DG (C36:4) in the region *m/z* 200–300 in which it was possible to detect under the specific collision energy employed (30 eV) the fragments corresponding to the protonated dehydrated FA. In the first case (Figure 4a), the isomer DG (C18:2/C18:0) was completely excluded because of the absence of both fatty acid fragments, then the *m/z* 603.53 corresponds only to the species (C18:1/C18:1); in the second case (Figure 4b), the presence of three different fragments of similar intensity in the MS/MS spectrum of the ion 599.50 *m/z* led to the identification of two DG species (C18:3/C18:1 and C18:2/C18:2), probably at similar concentrations; finally, in the third case the MS/MS spectrum of the ion 601.52 *m/z* (Figure 4c) shows three fragments of different intensity, then it corresponds to the mixture of two DGs (C18:2/C18:1 and C18:3/C18:0), but one of them (C18:2/C18:1) is significantly more abundant, while the other one (C18:3/C18:0) is present at very low level, even considering the absence of the C18:0 fragment.

**Figure 5:**
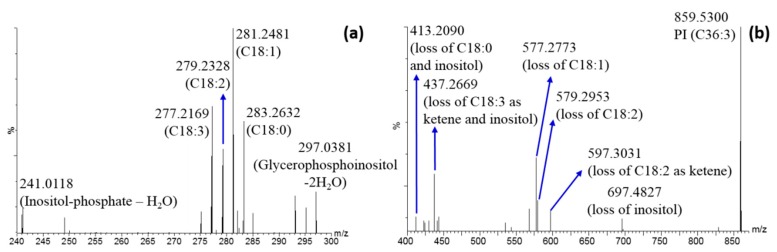
MS/MS spectrum of *m/z* 859.53 obtained in negative ionization mode at a collision energy of 30 eV: (**a**) over the range 240–300 *m/z*; (**b**) over the range 400–870 *m/z*.

Similar considerations were performed for PL structure elucidation. Their MS/MS spectra in negative ionization mode show diagnostic fragments in the FA region, helpful to select the right isomer; in addition, the PL class was properly confirmed by the detection of specific ion related to the polar head. As an example, Figure 5a reports the MS/MS spectra of phosphatidylinositol (PI) in the region 240–300 *m/z* containing fragment ions related to both fatty acids and the inositol polar group. Four fatty acids are present, meaning that the signal derives from two different PIs. According to Progenesis QI results, the *m/z* 859.5300 should correspond to PI (C36:3). In this case both PI (C18:2/C18:1) and PI (C18:3/C18:0) are present. In particular, looking at the ion intensity, the two isomers should be present at similar concentrations. The MS/MS spectrum in the region 400–700 *m/z* is informative, as well. It shows the losses of each fatty acid and /or inositol according to the scheme proposed in Figure 5b.

**Figure 6:**
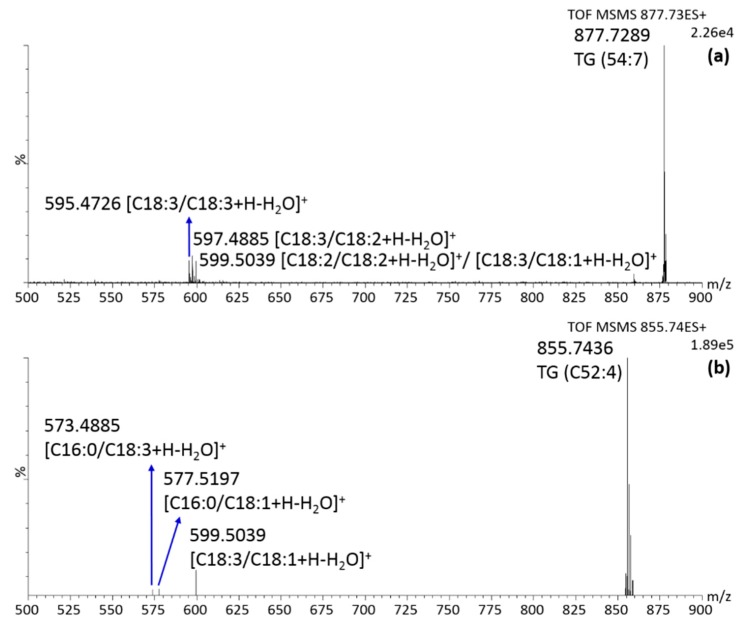
MS/MS spectrum in positive ionization mode over the range 500–900 *m/z* of (**a**) *m/z* 877.7289 and; (**b**) *m/z* 855.7436, obtained at a collision energy of 30 eV.

The identification of the TG was slightly more complicated due to the presence of another FA. However, also in this case, the high content of C18:1, C18:2 and C18:3 determined the choice of the most probable candidate. All the species for which MS/MS was necessary are marked with an asterisk in Table 3. Sometimes, more than one species was confirmed, meaning that more combinations are actually present in the sample. As an example, the MS/MS spectrum of *m/z* 877.7289 is shown in Figure 6a along with fragment elucidation. Several combinations of C18:1, C18:2 and C18:3 were detected in the DG region, then both TG (C18:1/C18:3/C18:3) and TG (C18:2/C18:2/C18:3) were positively identified and present at similar concentration levels. On the other hand, the interpretation of the MS/MS spectrum of *m/z* 855.7436, reported in Figure 6b, led to the rapid identification of the species TG (C18:3/C18:1/C16:0) as the most abundant among all the candidates, while the highly probable combination (C18:2/C18:2/C16:0) was excluded due to the absence of a signal at *m/z* 575, corresponding to the DG fragment (C18:2/C16:0). To conclude this section, a total of 61 compounds were positively identified in the *K. africana* fruit, the majority of them for the first time. The innovative analytical technique allowed to detect both polar (PLs and FAs) and non-polar lipids (MG, DG, and TG), without any chromatographic separation and/or sample preparation step, usually required in other analytical systems.

An approach similar to that used for phenol quantification was applied to lipids. Specifically, the percentages of acylglycerols were roughly estimated through the area percentages of the corresponding ions detected in positive mode, while FAs and PLs through the integration of the relative peaks in negative. As for acylglycerols, TGs represent more than 50% of the total and account for twice the DG species, which in turn are about three times the MG class. As for the polar fraction, it is mainly constituted of FAs (94%), while they are all present at percentages less than 1%. 

## 3. Materials and Methods 

### 3.1. Samples

The fruits (*n* = 2) of *Kigelia africana* were collected in the village of Gallè (Mali) in the autumn of two different years (2016 and 2018). The samples have been initially dried, subsequently shredded in fine powder. A conductive product was prepared by adding ultrapure water. 

### 3.2. Reagents and Material

Acetonitrile, 2-propanol, methanol, formic acid (all LC-MS grade), n-hexane and *N*,*N*-dimethylformamide (both reagent grade) were purchased from Merck Life Science (Merck KGaA, Darmstadt, Germany). Ultrapure water was obtained from Milli-Q advantage A10 system (Millipore, Bedford, MA, USA). Standards of isoquercetrin, 2-hydroxybenzoic acid, 4-hydroxybenzoic acid, vanillin, vanillin alcohol, p-coumaric acid, caffeic acid, daidzein, 1,3-benzenediol, syringol, homovanillic acid and hydroxytyrosol were acquired from Merck Life Science

### 3.3. Rapid Evaporative-Ionization Mass Spectrometry (REIMS) Instrumentation and Analytical Conditions

Analyses were carried out using a diathermy pencil sampling device equipped with a diathermy electrosurgical generator (Erbe VIO 50 C, Erbe, Tuebingen, Germany), which instantly vaporizes molecules from the sample. Two different high-frequency electrical current to the samples, forced-coagulation (FC) at a power of 20 W and dry-cut (DC) at 10 W, were applied during the analyses in positive and negative ionization mode, respectively.

Sampling was carried out 3 to 5 seconds to obtain an intense mass spectrum, technical replicates were analyzed, thus taking into account repeatability of the analysis. MS analyses were performed by a Xevo G2 XS QToF instrument, interfaced to a REIMS source, equipped with an helical coiled ribbon collision surface heated by a constant current power supply set to 4.5 A and 4.2 V (Kanthal D 1.0 × 0.1 mm) (Waters Corporation, Wilmslow, UK). The instrument was operated under negative and positive mode, mass spectra were recorded from *m/z* 100 to 1500 according to the molecular weight of the expected compounds. 

A solution of 2-propanol was infused into the REIMS source through a syringe pump (fixed luer lock LC pump priming syringe by Trajan Scientific, Crownhill, UK), at a constant flow rate of 150 µL min^−1^ to promote the ionization of different compounds and obtain a constant source of cleaning.

The instrument was calibrated prior to use by infusing sodium formate in 2-propanol via the matrix inlet on the REIMS source. The mass resolution was 25,000 FWHM over the mass range of interest. The cone voltage was set at 80 V. The endogenous ions *m/z* 281.2486 and *m/z* 855.7436, corresponding to the deprotonated molecule of octadecenoic acid (C_18_H_33_O_2_) and the protonated molecule of TG (C54:4) (C_55_H_99_O_6_), were used for internal lock-mass correction in negative and positive mode, respectively, after confirmation by MS/MS experiments. 

Precursor ions were isolated in the resolving quadrupole region of the instrument. Argon was used as the collision gas and collision energy was optimized for each compound. The following MS/MS specific settings were applied: LM and HR resolution 6.7 and 15.0, respectively, pre-filter 2.0 V and ion energy 0.8 V.

Data were acquired and visualized by using MassLynx v. 4.1 (Waters Corporation), and processed by Progenesis QI software version 2.3 (Nonlinear Dynamics, Newcastle, UK) through access to the online available Humane Metabolome Database, LipidMaps and Metlin platforms [33,34,35]. An automatic peak picking was performed in negative mode to generate a tentative assignments for each *m/z* value, while a minimum ion intensity of 500,000 was set in positive mode. A maximum mass error of 10 ppm was fixed. 

### 3.4. LC-PDA/MS Analysis of Phenolic Compounds of Kigelia africana (Lam) Benth.

The powder of the fruit (100 mg) was treated with 10 mL of hexane under continuous agitation for 10 min in order to remove the apolar fraction (lipids). After filtration, 10 mL of a methanol/water 60:40 (*v/v*) mixture were added to the solid residue and the mixture was sonicated for 30 min and centrifuged for 15 min at 1000 rpm; the pellet was extracted once again by using the same procedure and the two liquid phases were pooled. Then, the solvent was evaporated under vacuum and the extract was dissolved with 500 μL of a solution methanol/water/dimethylformamide (2:2:1 *v/v/v*), prior of the injection into a Nexera X2 system coupled to a PDA and LCMS-2020 mass spectrometer (Shimadzu, Kyoto, Japan). The separation was carried out on an Ascentis Express C18 column 150 × 4.6 mm (L × ID), 2.7 μm dp column (Merck Life Science), operated at 1 mL/min flow rate, split to 0.4 mL/min prior to MS detection. Water 0.1% formic acid (A) and Acetonitrile (B) were used as mobile phase, under the following gradient elution program: 0–5 min, 2% B; 20 min 10% B; 60 min 30% B; 80 min 100% B. Column oven was set at 30 °C, the injection volume was 10 μL.

## 4. Conclusions

The present research work provided a fast and comprehensive screening of the chemical constituents of *K. africana* fruits. Besides confirming the presence of antioxidant molecules such as flavonoids and iridoids, which were detected despite minor components, the major compounds were lipids. In particular, the native lipid composition was elucidated here for the first time and could address new studies about the biological activity of this plant, at the moment attributed only to phenol-like compounds. For instance, monounsaturated FAs could be involved in the regulation of cholesterol and triglyceride levels in the cells, while the high content of the polyunsaturated fatty acids in the glycerolipid and glycerophospholipid structures could be responsible for some beneficial activities against diabetes and cardiovascular disorders. Generally, the fruit investigated here could represent a rich source of essential FAs, such as ω-6 linoleic acid and ω-3 linolenic acid, both involved in numerous metabolic pathways, thus acting as signaling molecules. 

## Figures and Tables

**Table 1:** Average signal-to-noise (S/N) ratio and relative coefficient of variation (CV%) for 7 ions, at both intra- and interday (two days) levels. The interday CV% was calculated on two different samples.

*m/z*	Chemical Class	Ion Mode	Average S/N	CV% Intraday–Intrasamplen *n* = 3	CV% Interday–Intersamplen *n* = 12
281.2481	Fatty acid	-	160.85	7.88	8.27
487.0825	phenol	+	66.90	9.25	16.78
523.1470	iridoid	-	13.12	21.36	23.09
603.5354	diglyceride	+	24.33	0.56	14.54
725.5056	phospholipid	-	5.80	19.42	15.85
877.7289	triglyceride	+	86.39	15.16	18.97
915.6842	triglyceride	+	121.00	5.03	5.33

**Table 2:** Tentative identification of phenol-like compounds in the fruits of *Kigelia africana*, along with the kind of detected ion (deprotonated [M − H]^−^, adduct ion [M + Na]^+^ or [M + K]^+^ or adduct ion of dimer [2M + Na]^+^), its chemical formula and theoretical mass. The mass error (part per million, ppm) and the chemical class of each assignment are also provided.

Detected Ion (*m/z*)	Tentative Assignment	Detected Ion	Chemical Formula	Theoretical Mass	Mass Error (ppm)	Chemical Class	Area %
109.0291	Pyrocatechol	[M − H]^−^	C_6_H_5_O_2_	109.0295	−3.67	Phenol	1.64
137.0249	4-Hydroxybenzoic acid ^#^	[M − H]^−^	C_7_H_5_O_3_	137.0244	3.30	Phenol	20.98
141.0199	Kojic acid ^*^	[M − H]^−^	C_6_H_5_O_4_	141.0193	−4.25	Pyranone	0.33
151.0395	Vanillin ^#^	[M − H]^−^	C_8_H_7_O_3_	151.0395	0	Phenol	7.33
153.0556	Vanillin alcohol ^#^	[M − H]^−^	C_8_H_9_O_3_	153.0551	−3.26	Phenol	2.52
163.0399	p-coumaric acid *,^#^	[M − H]^−^	C_9_H_7_O_3_	163.0395	−2.45	Phenol	9.16
179.0347	Caffeic Acid *,^#^	[M − H]^−^	C_9_H_7_O_4_	179.0344	−1.67	Phenol	4.01
239.0704	Dehydro-α-lapochone ^*^	[M − H]^−^	C_15_H_11_O_3_	239.0708	−1.67	Quinone	8.14
241.0850	Lapachol *	[M − H]^−^	C_15_H_13_O_3_	241.0865	6.22	Quinone	4.09
253.0495	Daidzein ^#^	[M − H]^−^	C_15_H_9_O_4_	253.0500	1.98	Phenol	2.04
355.1037	1-*O*-Feruloylglucose	[M − H]^−^	C_16_H_19_O_9_	355.1035	−0.56	Phenol	5.00
383.1164	Piperenol A	[M − H]^−^	C_21_H_19_O_7_	383.1131	−8.61	Benzoate	30.76
523.1470	Verminoside *	[M − H]^−^	C_24_H_27_O_13_	523.1451	−3.63	Phenol/iridoid	4.01
TOTAL							100
383.0776	Rosmarinic acid ^*^	[M + Na]^+^	C_18_H_16_O_8_Na	383.0742	8.87	Phenol	9.25
475.0970	Epigallocatechin-p-coumaroate	[M + Na]^+^	C_24_H_20_O_9_Na	475.1005	−7.37	Phenol	60.91
487.0825	Isoquercetrin *,^#^	[M + Na]^+^	C_21_H_20_O_12_Na	487.0852	−5.54	Phenol	22.35
547.1375	Verminoside*	[M + Na]^+^	C_24_H_28_O_13_Na	547.1427	−9.50	Phenol/iridoid	7.49
TOTAL							100
491.0701	Epigallocatechin-p-coumaroate	[M + K]^+^	C_24_H_20_O_9_K	491.0744	−8.76	Phenol	8.98
549.1154	Piperenol A triacetate	[M + K]^+^	C_27_H_26_O_10_K	549.1163	1.64	Benzoate	66.66
563.1129	Verminoside *	[M + K]^+^	C_24_H_28_O_13_K	563.1167	−6.75	Phenol/iridoid	24.35
TOTAL							100
531.1043	Daidzein ^#^	[2M + Na]^+^	C_30_H_20_O_8_Na	531.1055	−2.26	Phenol	100

^*^ confirmed by the literature. ^#^ confirmed by LC-MS analysis and standard injection

**Table 3:** Tentative identification of lipid compounds in the fruits of *Kigelia africana*, along with the kind of detected ion (deprotonated [M − H]^−^ or dehydrated molecule [M + H − H_2_O]^+^), its chemical formula and theoretical mass. The mass error (part per million, ppm) for each assignment is also provided.

Detected Ion (*m/z*)	Tentative Assignment	Detected Ion	Chemical Formula	Theoretical Mass	Mass Error (ppm)	Area%
227.2012	C14:0	[M − H]^−^	C_14_H_27_O_2_	227.2011	−0.44	0.26
253.2173	C16:1	[M − H]^−^	C_16_H_29_O_2_	253.2167	−2.34	0.33
255.2325	C16:0	[M − H]^−^	C_16_H_31_O_2_	255.2324	−0.91	11.51
267.2319	C17:1	[M − H]^−^	C_17_H_31_O_2_	267.2324	1.87	0.62
269.2479	C17:0	[M − H]^−^	C_17_H_33_O_2_	269.2480	0.37	0.53
277.2169	C18:3	[M − H]^−^	C_18_H_29_O_2_	277.2167	−0.72	19.74
279.2328	C18:2	[M − H]^−^	C_18_H_31_O_2_	279.2324	−1.43	22.91
281.2481	C18:1	[M − H]^−^	C_18_H_33_O_2_	281.2480	−0.35	27.79
283.2632	C18:0	[M − H]^−^	C_18_H_35_O_2_	283.2637	1.76	5.29
297.2429	C18:1OH	[M − H]^−^	C_18_H_33_O_3_	297.2435	2.02	1.02
309.2791	C20:1	[M − H]^−^	C_20_H_37_O_2_	309.2793	0.65	0.37
311.2950	C20:0	[M − H]^−^	C_20_H_39_O_2_	311.2950	0	1.02
325.3114	C21:0	[M − H]^−^	C_21_H_41_O_2_	325.3106	−2.46	1.70
339.3252	C22:0	[M − H]^−^	C_22_H_43_O_2_	339.3263	3.24	0.92
TOTAL FAs						93.99
671.4620	PA (C34:2)	[M − H]^−^	C_37_H_68_O_8_P	671.4657	−5.51	0.45
673.4767	PA (C34:1)	[M − H]^−^	C_37_H_70_O_8_P	673.4814	−6.97	0.25
685.4788	PA (C35:2)	[M − H]^−^	C_38_H_70_O_8_P	685.4814	−3.79	0.19
693.4537	PA (C136:5)	[M − H]^−^	C_39_H_66_O_8_P	693.4501	5.19	0.07
695.4607	PA (C18:3/18:1 + C18:2/C18:2) ^#^	[M − H]^−^	C_39_H_68_O_8_P	695.4657	−7.18	0.29
697.4779	PA (C18:2/18:1 + C18:3/C18:0) ^#^	[M − H]^−^	C_39_H_70_O_8_P	697.4814	−5.02	0.75
699.4944	PA (C18:1/C18:1) ^#^	[M − H]^−^	C_39_H_72_O_8_P	699.4970	−3.72	0.75
723.4934	PA(C20:1/C18:3) ^#^	[M − H]^−^	C_41_H_72_O_8_P	723.4970	−4.97	0.35
725.5056	PA (C38:3)	[M − H]^−^	C_41_H_74_O_8_P	725.5127	−9.78	0.35
727.5222	PA (C38:2)	[M − H]^−^	C_41_H_76_O_8_P	727.5283	−8.38	0.22
742.5382	PE (C18:1/C18:1) ^#^	[M − H]^−^	C_41_H_77_NO_8_P	742.5392	−1.34	0.26
747.5159	PG (C34:1)	[M − H]^−^	C_40_H_76_O_10_P	747.5176	2.27	0.21
749.5262	PG (C34:0)	[M − H]^−^	C_40_H_78_O_10_P	749.5332	9.34	0.22
831.4979	PI (C34:3)	[M + H]^−^	C_43_H_76_O_13_P	831.5023	5.29	0.08
833.5137	PI (C34:2)	[M − H]^−^	C_43_H_78_O_13_P	833.5180	5.16	0.61
835.5310	PI (C34:1)	[M − H]^−^	C_43_H_80_O_13_P	835.5336	3.11	0.12
857.5195	PI (C18:3/18:1 + C18:2/C18:2) ^#^	[M − H]^−^	C_45_H_78_O_13_P	857.5180	−1.75	0.07
859.5300	PI (18:2/C18:1 + C18:3/C18:0) ^#^	[M − H]^−^	C_45_H_80_O_13_P	859.5336	4.19	0.30
861.5469	PI (C18:2/C18:1) ^#^	[M − H]^−^	C_45_H_82_O_13_P	861.5493	2.78	0.47
TOTAL PLs						6.01
TOTAL (Fas + PLs)						100
313.2746	MG (C16:0)	[M + H − H_2_O]^+^	C_19_H_37_O_3_	313.2737	2.87	1.77
335.2592	MG (C18:3)	[M + H − H_2_O]^+^	C_21_H_35_O_3_	335.2581	3.28	2.71
337.2744	MG (C18:2)	[M + H − H_2_O]^+^	C_21_H_37_O_3_	337.2737	2.07	2.41
339.2898	MG (C18:1)	[M + H − H_2_O]^+^	C_21_H_39_O_3_	339.2894	1.18	2.61
341.3053	MG (C18:0)	[M + H − H_2_O]^+^	C_21_H_41_O_3_	341.3050	0.88	1.48
TOTAL MGs						10.98
573.4885	DG (C34:3)	[M + H − H_2_O]^+^	C_37_H_65_O_4_	573.4883	−0.35	1.32
575.5036	DG (C34:2)	[M + H − H_2_O]^+^	C_37_H_67_O_4_	575.5040	0.69	1.89
577.5197	DG (C34:1)	[M + H − H_2_O]^+^	C_37_H_69_O_4_	577.5196	−0.17	3.09
579.5328	DG (C34:0)	[M + H − H_2_O]^+^	C_37_H_71_O_4_	579.5353	4.31	2.46
595.4726	DG (C36:6)	[M + H − H_2_O]^+^	C_39_H_63_O_4_	595.4727	0.17	1.75
597.4885	DG (C36:5)	[M + H − H_2_O]^+^	C_39_H_65_O_4_	597.4883	−0.33	3.02
599.5039	DG (C18:3/C18:1 + C18:2/C18:2) ^#^	[M + H − H_2_O]^+^	C_39_H_67_O_4_	599.5040	0.17	4.25
601.5198	DG (C18:2/C18:1 + C18:3/C18:0) ^#^	[M + H − H_2_O]^+^	C_39_H_69_O_4_	601.5195	0.50	3.92
603.5354	DG (C18:1/C18:1) ^#^	[M + H − H_2_O]^+^	C_39_H_71_O_4_	603.5352	−0.33	3.41
605.5507	DG (C18:0/C18:1) ^#^	[M + H − H_2_O]^+^	C_39_H_73_O_4_	605.5508	0.16	1.57
607.5642	DG (C18:0/C18:0) ^#^	[M + H − H_2_O]^+^	C_39_H_75_O_4_	607.5666	3.95	2.68
TOTAL DGs						29.36
851.7137	TG (C52:6)	[M + H]^+^	C_55_H_95_O_6_	851.7128	1.06	2.66
853.7281	TG (C52:5)	[M + H]^+^	C_55_H_97_O_6_	853.7285	−0.46	3.90
855.7436	TG (C18:3/C18:1/C16:0) ^#^	[M + H]^+^	C_55_H_99_O_6_	855.7436	0	3.42
857.7581	TG (C52:3)	[M + H]^+^	C_55_H_101_O_6_	857.7598	−1.98	1.66
859.7742	TG (C52:0)	[M + H]^+^	C_55_H_103_O_6_	859.7754	−1.39	0.47
873.6983	TG (C54:9)	[M + H]^+^	C_57_H_93_O_6_	873.6972	1.25	5.15
875.7136	TG (C54:8)	[M + H]^+^	C_57_H_95_O_6_	875.7128	0.91	8.40
877.7289	TG (C18:2/C18:2/C18:3 + C18:3/C18:3/C18:1) ^#^	[M + H]^+^	C_57_H_97_O_6_	877.7285	0.45	11.56
879.7445	TG (C18:2/C18:1/C18:3 + C18:2/C18:2/C18:2) ^#^	[M + H]^+^	C_57_H_99_O_6_	879.7441	0.45	10.45
881.7601	TG (C18:3/C18:1/C18:1 + C18:3/C18:2/C18:0) ^#^	[M + H]^+^	C_57_H_101_O_6_	881.7598	0.34	7.35
883.7756	TG (C18:1/C18:1/C18:2) ^#^	[M + H]^+^	C_57_H_103_O_6_	883.7754	0.23	3.38
885.7910	TG (C18:1/C18:1/C18:1) ^#^	[M + H]^+^	C_57_H_105_O_6_	885.7911	−0.11	1.26
TOTAL TGs						59.65
TOTAL (MGs + DGs + TGs)						100

^#^ Confirmed by MS/MS experiments

## Supplementary Materials

The following are available online at https://www.mdpi.com/1420-3049/25/4/962/s1: **Table S1**: S/N comparison of 5 representative ions detected in negative ionization mode between two different power of cutting (DC 10 W and FC 20 W), along with their coefficient of variation (CV%). **Table S2**: S/N comparison of 7 representative ions detected in positive ionization mode between two different power of cutting (DC 10 W and FC 20 W), along with their coefficient of variation (CV%). **Figure S1**: MS/MS spectrum over the range 50–300 of *m/z* 109.02 (pyrocatechol) obtained in negative ionization mode at a collision energy of 20 eV, by cutting the sample at DC 10 W. The ion at m/z 91.01 corresponds to the loss of water, while the ion at m/z 81.03 corresponds to the loss of ethylene and opening of the aromatic ring. **Figure S2**: MS/MS spectrum over the range 50–300 of *m/z* 137.01 (4-hydroxybenzoic acid) obtained in negative ionization mode at a collision energy of 20 eV, by cutting the sample at DC 10 W. The ion at *m/z* 93.03 corresponds to the loss of the carboxyl group, while the ion at *m/z* 76.98 corresponds to the further removal of the hydroxyl group. **Figure S3**: MS/MS spectrum over the range 80–300 of *m/z* 141.01 (kojic acid) obtained in negative ionization mode at a collision energy of 20 eV, by cutting the sample at DC 10 W. The ion at *m/z* 123.04 corresponds to the loss of water, while the ion at *m/z* 113.02 corresponds to the loss of a carbonyl group after the opening of the pyranone ring and *m/z* 95.01 corresponds to the loss of the –CH_2_OH chain followed by the opening of the ring and the loss of an additional alkyl group. **Figure S4**: MS/MS spectrum over the range 90–325 of *m/z* 151.03 (vanillin) obtained in negative ionization mode at a collision energy of 30 eV, by cutting the sample at DC 10 W. The ion at *m/z* 136.00 corresponds to the loss of the methyl group, the ion at *m/z* 123.04 corresponds to the loss of the carbonyl group, the ion at *m/z* 108.01 corresponds to the loss of both the methyl and the carbonyl group and the ion at *m/z* 93.03 corresponds to the deprotonated phenol. **Figure S5**: MS/MS spectrum over the range 90–325 of *m/z* 153.04 (vanillin alcohol) obtained in negative ionization mode at a collision energy of 20 eV, by cutting the sample at DC 10 W. The ion at *m/z* 138.00 corresponds to the loss of the methyl group, the ion at *m/z* 135.04 is the loss of water, the ion at *m/z* 125.04 corresponds to the loss of a carbonyl group (followed by rearrangement) and the ion at *m/z* 93.03 corresponds to the deprotonated phenol. **Figure S6**: MS/MS spectrum over the range 150–550 of *m/z* 241.03 (lapachol) obtained in negative ionization mode at a collision energy of 20 eV, by cutting the sample at DC 10 W. The ion at *m/z* 226.05 corresponds to the loss of the methyl group, the ion at *m/z* 213.04 corresponds to the loss of ethylene from the side chain, the ion at *m/z* 197.04 could be generated by the simultaneous loss of ethylene and a hydroxyl group, the ion at *m/z* 185.05 is probably related to the cleavage of the side chain and the ion at *m/z* 157.05 corresponds to the deprotonated naphtoquinone. **Figure S7**: MS/MS spectrum over the range 190–550 of *m/z* 253.07 (daidzein) obtained in negative ionization mode at a collision energy of 20 eV, by cutting the sample at DC 10 W. The ion at *m/z* 235.06 corresponds to the loss of water, while the ion at *m/z* 225.04 is the loss of the carbonyl group after the opening of the pyranone ring. **Figure S8**: MS/MS spectrum over the range 100–500 of *m/z* 487.08 obtained in positive ionization mode at a collision energy of 30 eV, by cutting the sample at FC 20 W. **Figure S9**: MS/MS spectrum over the range 50–1200 of *m/z* 523.14 obtained in negative ionization mode at a collision energy of 30 eV, by cutting the sample at DC 10 W.

## References

[1] Cragg G.M., Newman D.J., Snader K.M. (1997). Natural products in drug discovery and development. J. Nat. Prod..

[2] WHO (2002). WHO Guidelines on Good Agricultural and Collection Practices (GACP) for Medicinal Plants.

[3] Oladele A.T., Alade G.O., Omobuwajo O.R. (2011). Medicinal plants conservation and cultivation by traditional medicine practitioners (TMPs) in Aiyedaade Local Government Area of Osun State, Nigeria. Agric. Biol. J. N. Am..

[4] Azu O.O., Duru F.I.O., Osinubi A.A., Oremosu A.A., Norohna C.C., Okanlawon A.O., Elesha S.O. (2011). Long-term treatment with *Kigelia africana* fruit extract ameliorates the testicular toxicity following cisplatin administration in male Sprague-Dawley rats. J. Med. Plants Res..

[5] Osmana A.G., Ali Z., Chittiboyina A.G., Khan I.A. (2017). *Kigelia africana* Fruit: Constituents, Bioactivity, and Reflection on Composition Disparities. World J. Tradit. Chin. Med..

[6] Olaleye M.T., Rocha J.B. (2007). Commonly used tropical medicinal plants exhibit distinct in vitro antioxidant activities against hepatotoxins in rat liver. Exp. Toxical. Pathol..

[7] Akunyili D.N., Houghton P.J. (1993). Meroterpenoids and naphthaquinones from *Kigelia pinnata*. Phytochemistry.

[8] Kolodziej H. (1997). Protective role of *Kigelia africana* fruits against benzo(a) pyrene induced fore-stomach tumourigenesis in mice and against albumen induced inflammation in rates. Pharmacol. Lett..

[9] El-Sayyad S.M. (1981). Flavonoids of the leaves and fruits of Kigelia pinnata. Fitoterapia.

[10] Khan M.R., Mlungwana S.M. (1999). γ-Sitosterol, a cytotoxic sterol from *Markhamia zanzibarica* and *Kigelia africana*. Fitoterapia.

[11] Gouda Y.G., Abdel-baky A.M., Darwish F.M., Mohamed K.M., Kasai R., Yamasaki K. (2003). Iridoids from *Kigelia pinnata* DC. Fruits. Phytochemistry.

[12] Jabeen B., Riaz N. (2013). Isolation and characterization of limonoids from *Kigelia africana*. Z. Nat..

[13] Balog J., Sasi-Szabó L., Kinross J., Lewis M.R., Muirhead L.J., Veselkov K., Mirnezami R., Dezső B., Damjanovich L., Darzi A. (2013). Intraoperative tissue identification using rapid evaporative ionization mass spectrometry. Sci. Transl. Med..

[14] Alexander J., Gildea L., Balog J., Speller A., McKenzie J., Muirhead L., Scott A., Kontovounisios C., Rasheed S., Teare J. (2016). A novel methodology for in vivo endoscopic phenotyping of colorectal cancer based on real-time analysis of the mucosal lipidome: A prospective observational study of the iKnife. Surg. Endosc..

[15] Phelps D.L., Balog J., Gildea L.F., Bodai Z., Savage A., El-Bahrawy M.A., Speller A.V.M., Rosini F., Kudo H., McKenzie J.S. (2018). The surgical intelligent knife distinguishes normal, borderline and malignant gynaecological tissues using rapid evaporative ionisation mass spectrometry (REIMS). Br. J. Cancer.

[16] Rigano F., Stead S., Mangraviti D., Jandova R., Petit D., Marino N., Mondello L. (2019). Use of an “Intelligent Knife” (iknife), Based on the Rapid Evaporative Ionization Mass Spectrometry Technology, for Authenticity Assessment of Pistachio Samples. Food Anal. Methods.

[17] Shen Q., Li L., Song G., Feng J., Li S., Wang Y., Ma J., Wang H. (2020). Development of an intelligent surgical knife rapid evaporative ionization mass spectrometry based method for real-time differentiation of cod from oilfish. J. Food Compos. Anal..

[18] Balog J., Szaniszlo T., Schaefer K.C., Denes J., Lopata A., Godorhazy L., Szalay D., Balogh L., Sasi-Szabo L., Toth M. (2010). Identification of Biological Tissues by Rapid Evaporative Ionization Mass Spectrometry. Anal. Chem..

[19] Genangeli M., Heeren R.M.A., Porta Siegel T. (2019). Tissue classification by rapid evaporative ionization mass spectrometry (REIMS): Comparison between a diathermic knife and CO2 laser sampling on classification performance. Anal. Bioanal. Chem..

[20] Balog J., Kumar S., Alexander J., Golf O., Huang J., Wiggins T., Abbassi-Ghadi N., Enyedi A., Kacska S., Kinross J. (2015). In Vivo Endoscopic Tissue Identification by Rapid Evaporative Ionization Mass Spectrometry (REIMS). Angew. Chem. Int. Ed..

[21] Balog J., Perenyi D., Guallar-Hoyas C., Egri A., Pringle S.D., Stead S., Chevallier O.P., Elliott C.T., Takats Z. (2016). Identification of the Species of Origin for Meat Products by Rapid Evaporative Ionization Mass Spectrometry. J. Agric. Food Chem..

[22] Verplanken K., Stead S., Jandova R., Van Poucke C., Claereboudt J., Vanden Bussche J., De Saeger S., Takats Z., Wauters J., Vanhaecke L. (2017). Rapid evaporative ionization mass spectrometry for high-throughput screening in food analysis: The case of boar taint. Talanta.

[23] Black C., Chevallier O.P., Haughey S.A., Balog J., Stead S., Pringle S.D., Riina M.V., Martucci F., Acutis P.L., Morris M. (2017). A real time metabolomic profiling approach to detecting fish fraud using rapid evaporative ionisation mass spectrometry. Metabolomics.

[24] Lin Y., Wang H., Rao W., Cui Y., Yu X., Dai Z., Shen Q. (2018). Rapid Evaporative Ionization Mass Spectrometry-Based Lipidomics Tracking of Grass Carp (Ctenopharyngodon idellus) during In Vitro Multiple-Stage Digestion. J. Agric. Food Chem..

[25] Rigano F., Mangraviti D., Stead S., Martin N., Petit D., Dugo P., Mondello L. (2019). Rapid evaporative ionization mass spectrometry coupled with an electrosurgical knife for the rapid identification of Mediterranean Sea species *Anal*. Bioanal. Chem..

[26] Shen Q., Wang J., Li S., Rao W., Wang Y., Wang H. (2019). In Situ rapid evaporative ionization mass spectrometry method for real-time discrimination of Pelodiscus sinensis in different culturing modes without sample preparation. Food Anal. Methods.

[27] Strittmatter N., Rebec M., Jones E.A., Golf O., Abdolrasouli A., Balog J., Behrends V., Veselkov K.A., Takats Z. (2014). Characterization and Identification of Clinically Relevant Microorganisms Using Rapid Evaporative Ionization Mass Spectrometry. Anal. Chem..

[28] Golf O., Strittmatter N., Karancsi T., Pringle S.D., Speller A.V.M., Mroz A., Kinross J.M., Abbassi-Ghadi N., Jones E.A., Takats Z. (2015). Rapid Evaporative Ionization Mass Spectrometry Imaging Platform for Direct Mapping from Bulk Tissue and Bacterial Growth Media. Anal. Chem..

[29] Bolt F., Cameron S.J.S., Karancsi T., Simon D., Schaffer R., Rickards T., Hardiman K., Burke A., Bodai Z., Perdones-Montero A. (2016). Automated High-Throughput Identification and Characterization of Clinically Important Bacteria and Fungi using Rapid Evaporative Ionization Mass Spectrometry. Anal. Chem..

[30] Bodai Z., Cameron S., Bolt F., Simon D., Schaffer R., Karancsi T., Balog J., Rickards T., Burke A., Hardiman K. (2018). Effect of Electrode Geometry on the Classification Performance of Rapid Evaporative Ionization Mass Spectrometric (REIMS) Bacterial Identification. J. Am. Soc. Mass Spectrom..

[31] Golf O., Muirhead L.J., Speller A., Balog J., Abbassi-Ghadi N., Kumar S., Mróz A., Veselkov K., Takáts Z. (2015). XMS: Cross-Platform Normalization Method for Multimodal Mass Spectrometric Tissue Profiling. J. Am. Soc. Mass Spectrom..

[32] St John E.R., Balog J., McKenzie J.S., Rossi M., Covington A., Muirhead L., Bodai Z., Rosini F., Speller A.V.M., Shousha S. (2017). Rapid evaporative ionisation mass spectrometry of electrosurgical vapours for the identification of breast pathology: Towards an intelligent knife for breast cancer surgery. Breast Cancer Res..

[33] Wagh M.S., Wani S.B. (2019). Gastrointestinal Interventional Endoscopy: Advanced Techniques.

[34] Human Metabolome Database. http://www.hmdb.ca/.

[35] Metlin. https://metlin.scripps.edu/landing_page.php?pgcontent=mainPage.

[36] LIPID MAPS Lipidomics Gateway. https://www.lipidmaps.org/.

[37] Picerno P., Autore G., Marzocco S., Meloni M., Sanogo R., Aquino R.P. (2005). Anti-inflammatory Activity of Verminoside from *Kigelia africana* and Evaluation of Cutaneous Irritation in Cell Cultures and Reconstituted Human Epidermis. J. Nat. Prod..

[38] Bello I., Shehu M.W., Musa M., Asmawi M.Z., Mahmud R. (2016). *Kigelia africana* (Lam) Benth. (Sausage tree): Phytochemistry and pharmacological review of a quintessential African traditional medicinal plant. J. Ethnopharmacol..

[39] Hussain T., Fatima I., Rafay M., Shabir S., Akram M., Bano S. (2016). Evaluation of antibacterial and antioxidant activity of leaves, fruit and bark of *Kigelia africana*. Pak. J. Bot..

[40] Micheli V., Sanogo R., Mobilia M.A., Occhiuto F. (2019). Effects of *Kigelia africana* (Lam.) Benth. Fruits extract on the development and maturation of the reproductive system in immature male rats. Nat. Prod. Res..

[41] Costa R., Albergamo A., Pellizzeri V., Dugo G. (2016). Phytochemical screening by LC-MS and LC-PDA of ethanolic extracts from the fruits of *Kigelia africana* (Lam.) Benth. Nat. Prod. Res..

[42] Falode J.A., Obafemi T.O., Akinmoladun A., Olaleye M., Boligon A., Athayde M.L. (2016). High-Performance Liquid Chromatography (HPLC) Fingerprinting and Comparative Antioxidant Properties of Fruit and Leaf Extracts of *Kigelia africana*. Int. J. Pharm. Phytochem. Res..

[43] Sidjui L., Toghueo R.M.K., Zeuko’ E.M., Mbouna C.D.J., Mahiou-Leddet V., Herbette G., Fekam F.B., Ollivier E., Folefoc G.N. (2016). Antibacterial activity of the crude extracts, fractions and compounds from the stem barks of Jacaranda mimosifolia and *Kigelia africana* (Bignoniaceae). Pharmacologia.

[44] Gomes M.N., Augustine T.N., Moyoa D., Chivandi E. (2019). Differential response of breast cancer cell lines to *Kigelia africana*, Ximenia caffra and Mimusops zeyheri seed oils. S. Afr. J. Bot..

