# Supplementary material for: Exploration of Rapid Evaporative-Ionization Mass Spectrometry as a Shotgun Approach for the Comprehensive Characterization of Kigelia Africana (Lam) Benth. Fruit
Source: Molecules. 2020 Feb 20;25(4):962. doi: 10.3390/molecules25040962 (PMC7070896; doi:10.3390/molecules25040962)
Supplement: Supplementary file 1 [file molecules-25-00962-s001.pdf]

Supplementary material

Exploration of Rapid Evaporative-Ionization Mass Spectrometry as a Shotgun Approach for the Comprehensive Characterization of *Kigelia Africana* (Lam) Benth. Fruit

Katia Arena [1,2,], Francesca Rigano [2,*], Domenica Mangraviti [2], Francesco Cacciola [3], Francesco Occhiuto [2], Laura Dugo [4], Paola Dugo [2,5] and Luigi Mondello [2,4,5,6]

[1] Foundation A. Imbesi c/o University of Messina, Messina, Italy; arenak@unime.it
[2] Department of Chemical, Biological, Pharmaceutical and Environmental Sciences, University of Messina, I-98168 Messina, Italy; dmangraviti@unime.it (D.M.); focchiuto@unime.it (F.O.); pdugo@unime.it (P.D.); lmondello@unime.it (L.M.)
[3] Department of Biomedical, Dental, Morphological and Functional Imaging Sciences, University of Messina, I-98168 Messina, Italy; cacciolaf@unime.it
[4] Department of Sciences and Technologies for Human and Environment, University Campus Bio-Medico of Rome, I-00128 Rome, Italy; l.dugo@unicampus.it
[5] Chromaleont s.r.l., c/o Department of Chemical, Biological, Pharmaceutical and Environmental Sciences, University of Messina, I-98168 Messina, Italy
[6] BeSep s.r.l., c/o Department of Chemical, Biological, Pharmaceutical and Environmental Sciences, University of Messina, I-98168 Messina, Italy
* Correspondence: frigano@unime.it

Table S1. S/N comparison of 5 representative ions detected in negative ionization mode between two different power of cutting (DC 10 W and FC 20 W), along with their coefficient of variation (CV%).

m/z	tentative assignment	DC 10 W		FC 20 W	
		S/N	CV%	S/N	CV%
137.0249	4-hydroxybenzoic acid	34.77	10.21	12.72	16.87
281.2486	Octadecenoic acid (C18:1)	160.85	7.88	67.14	4.35
523.147	Verminoside	13.12	21.36	9.96	21.04
671.462	PA (C34:2)	10.18	6.78	-	
861.5469	PI (C18:2/C18:1)	5.92	14.59	-	

Table S2. S/N comparison of 7 representative ions detected in positive ionization mode between two different power of cutting (DC 10 W and FC 20 W), along with their coefficient of variation (CV%).

m/z	tentative assignment	DC 10 W		FC 20 W	
		S/N	CV%	S/N	CV%
339.2898	MG (C18:1)	90.15	11.41	25.51	3.81
383.0776	Rosmarinic acid	-		10.53	19.87
475.0970	Epigallocatechin-p-coumaroate	-		146.49	11.81
549.1154	Piperenol A triacetate	-		67.32	2.99
599.5039	DG (C36:4)	43.88	3.70	54.41	16.60
853.7281	TG (52:5)	11.43	4.06	32.86	13.99
877.7289	TG (54:7)	25.21	18.00	86.39	15.16

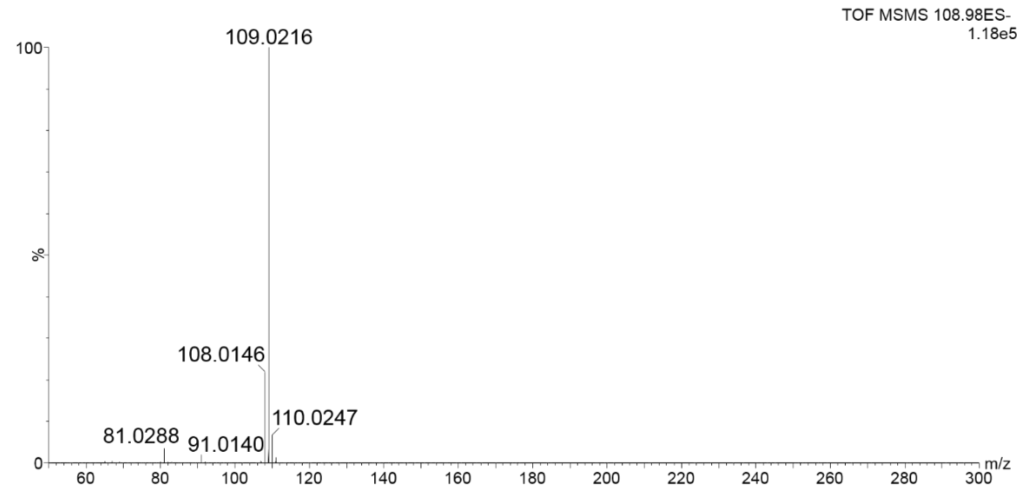

Figure S1. MS/MS spectrum over the range 50-300 of m/z 109.02 (pyrocatechol) obtained in negative ionization mode at a collision energy of 20 eV, by cutting the sample at DC 10 W. The ion at *m/z* 91.01 corresponds to the loss of water, while the ion at *m/z* 81.03 corresponds to the loss of ethylene and opening of the aromatic ring.

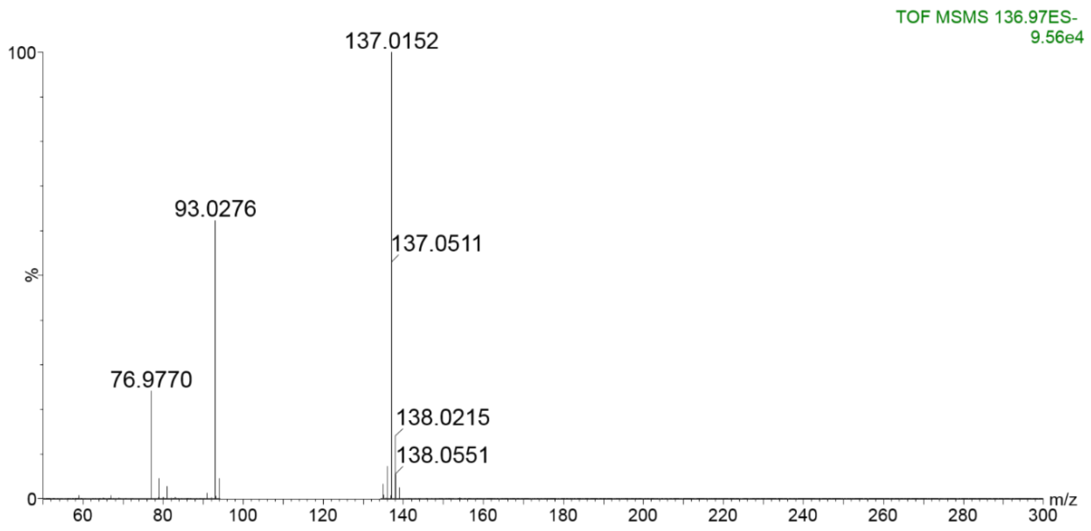

Figure S2. MS/MS spectrum over the range 50-300 of m/z 137.01 (4-hydroxybenzoic acid) obtained in negative ionization mode at a collision energy of 20 eV, by cutting the sample at DC 10 W. The ion at *m/z* 93.03 corresponds to the loss of the carboxyl group, while the ion at *m/z* 76.98 corresponds to the further removal of the hydroxyl group.

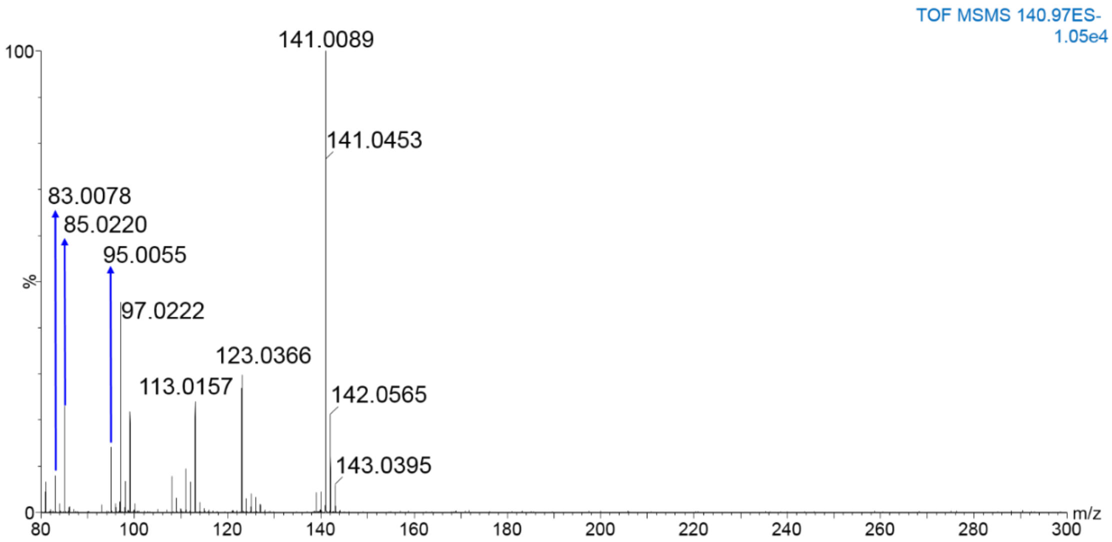

Figure S3. MS/MS spectrum over the range 80-300 of m/z 141.01 (kojic acid) obtained in negative ionization mode at a collision energy of 20 eV, by cutting the sample at DC 10 W. The ion at *m/z* 123.04 corresponds to the loss of water, while the ion at *m/z* 113.02 corresponds to the loss of a carbonyl group after the opening of the pyranone ring and *m/z* 95.01 corresponds to the loss of the $-CH_2OH$ chain followed by the opening of the ring and the loss of an additional alkyl group.

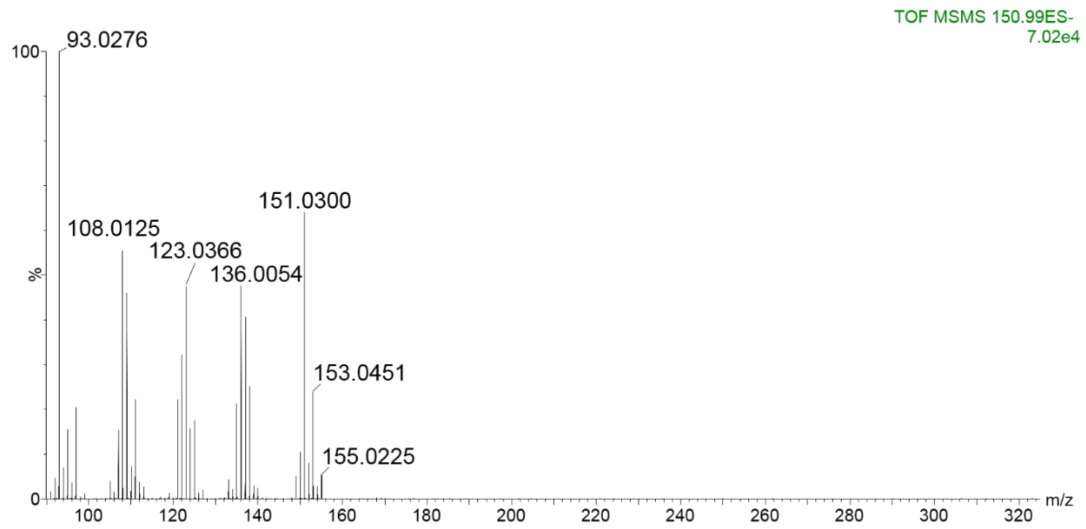

Figure S4. MS/MS spectrum over the range 90-325 of m/z 151.03 (vanillin) obtained in negative ionization mode at a collision energy of 30 eV, by cutting the sample at DC 10 W. The ion at *m/z* 136.00 corresponds to the loss of the methyl group, the ion at *m/z* 123.04 corresponds to the loss of the carbonyl group, the ion at *m/z* 108.01 corresponds to the loss of both the methyl and the carbonyl group and the ion at *m/z* 93.03 corresponds to the deprotonated phenol.

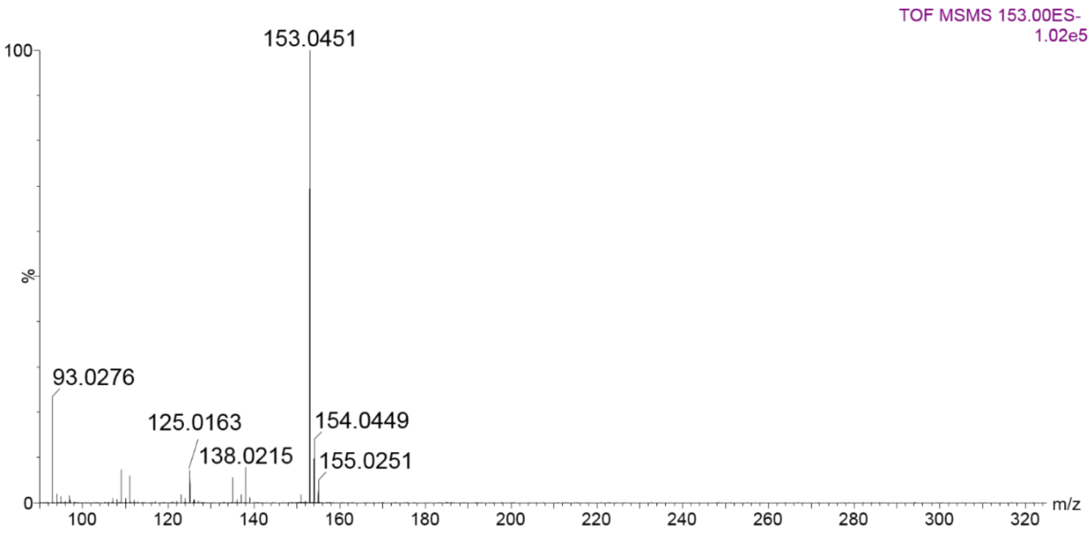

Figure S5. MS/MS spectrum over the range 90-325 of m/z 153.04 (vanillin alcohol) obtained in negative ionization mode at a collision energy of 20 eV, by cutting the sample at DC 10 W. The ion at *m/z* 138.00 corresponds to the loss of the methyl group, the ion at *m/z* 135.04 is the loss of water, the ion at *m/z* 125.04 corresponds to the loss of a carbonyl group (followed by rearrangement) and the ion at *m/z* 93.03 corresponds to the deprotonated phenol.

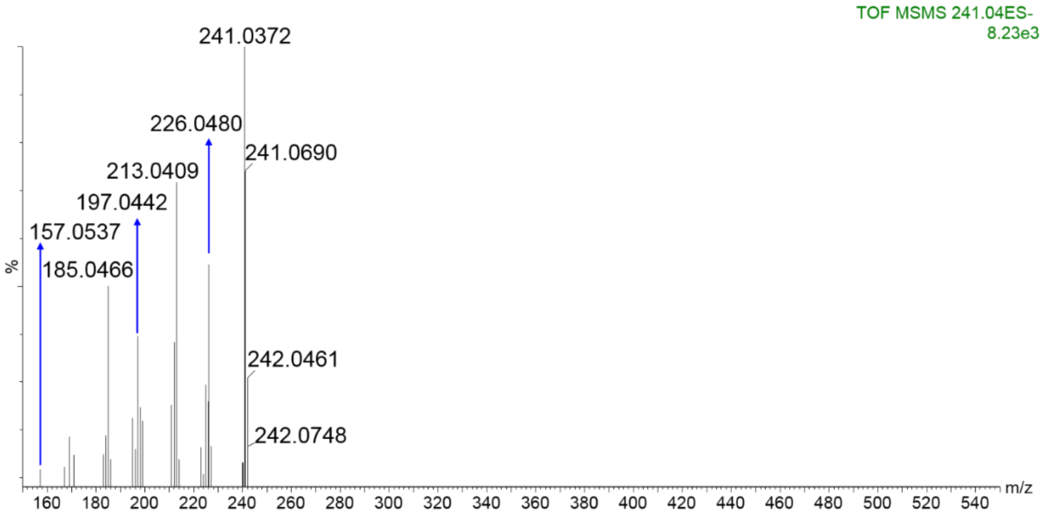

Figure S6. MS/MS spectrum over the range 150-550 of m/z 241.03 (lapachol) obtained in negative ionization mode at a collision energy of 20 eV, by cutting the sample at DC 10 W. The ion at *m/z* 226.05 corresponds to the loss of the methyl group, the ion at *m/z* 213.04 corresponds to the loss of ethylene from the side chain, the ion at *m/z* 197.04 could be generated by the simultaneous loss of ethylene and a hydroxyl group , the ion at *m/z* 185.05 is probably related to the cleavage of the side chain and the ion at at *m/z* 157.05 corresponds to the deprotonated naphtoquinone.

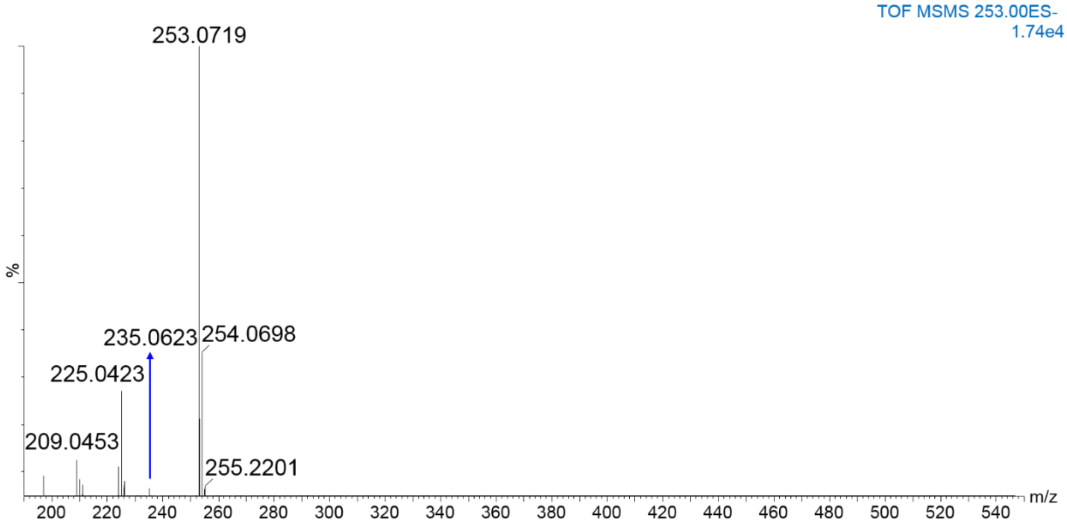

Figure S7. MS/MS spectrum over the range 190-550 of m/z 253.07 (daidzein) obtained in negative ionization mode at a collision energy of 20 eV, by cutting the sample at DC 10 W. The ion at *m/z* 235.06 corresponds to the loss of water, while the ion at *m/z* 225.04 is the loss of the carbonyl group after the opening of the pyranone ring.

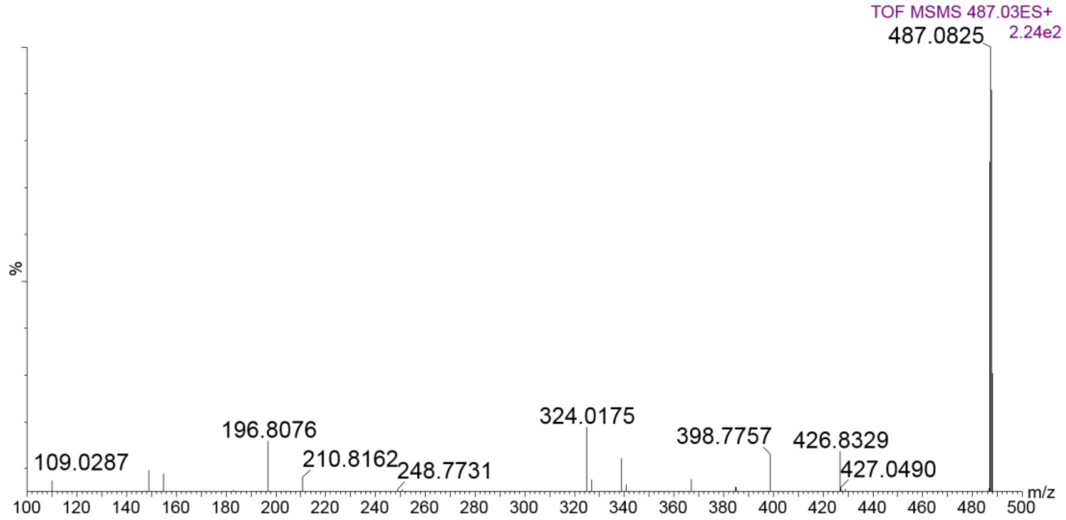

Figure S8. MS/MS spectrum over the range 100-500 of m/z 487.08 obtained in positive ionization mode at a collision energy of 30 eV, by cutting the sample at FC 20 W.

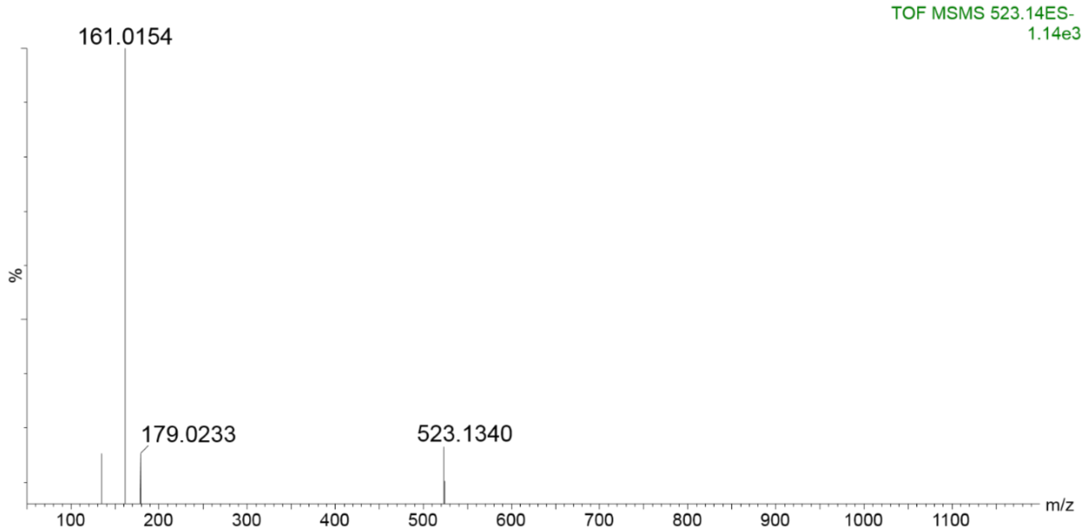

Figure S9. MS/MS spectrum over the range 50-1200 of m/z 523.14 obtained in negative ionization mode at a collision energy of 30 eV, by cutting the sample at DC 10 W.
